# Treatment success in cats with chronic enteropathy is associated with a decrease in fecal calprotectin concentrations

**DOI:** 10.3389/fvets.2024.1390681

**Published:** 2024-04-03

**Authors:** Romy M. Heilmann, Denise S. Riggers, Isla Trewin, Gábor Köller, Aarti Kathrani

**Affiliations:** ^1^Department for Small Animals, College of Veterinary Medicine, University of Leipzig, Leipzig, Germany; ^2^Department of Clinical Science and Services, Royal Veterinary College, London, United Kingdom; ^3^Department for Large Animals, College of Veterinary Medicine, University of Leipzig, Leipzig, Germany

**Keywords:** biomarker, diet-responsive, feline, immunomodulation, inflammation, therapeutic trial

## Abstract

Feline chronic enteropathies (FCE) are challenging to diagnose and monitor for progression and response to treatment. Fecal calprotectin might be a useful non-invasive marker to evaluate clinical endpoints of therapeutic monitoring in FCE. We evaluated fecal calprotectin concentrations in cats with FCE before and after initiation of treatment comprised of immunomodulation and/or dietary intervention. Included were 17 cats with FCE and 18 healthy controls. Clinical investigation of FCE cases included clinical severity grading (feline chronic enteropathy activity index, FCEAI) in all cats, abdominal ultrasonography in 15 cats, and gastrointestinal biopsies in 6 cats. Fecal calprotectin was measured in samples from 12 cats with FCE before treatment, all 17 FCE cats ≥6 weeks after treatment initiation, and all healthy controls. Fecal calprotectin concentrations in FCE cases before treatment (median: 61 μg/g) were significantly higher than after treatment initiation (median: 15 μg/g; *p* = 0.0098) and compared to controls (median: 6 μg/g; *p* = 0.0235) and correlated with the FCEAI scores (*ρ* = 0.54, *p* = 0.0316). Fecal calprotectin concentrations after treatment initiation were higher with more severe duodenal/proximal jejunal pathology (*ρ* = 0.83, *p* = 0.0427) and shorter intervals between sampling time points (*ρ* = −0.54, *p* = 0.0250). Relevant decreases in initially increased fecal calprotectin concentrations are seen in cats with FCE on varying treatment strategies that significantly improve or have remission of clinical signs. This supports the utility of fecal calprotectin as a surrogate biomarker to assess disease severity in FCE cases. Further studies need to evaluate fecal calprotectin concentrations longitudinally in relation to mucosal healing vs. clinical response.

## 1 Introduction

Chronic enteropathies (CE) in cats are commonly encountered in small animal veterinary practice (1, 2). Aside from the challenges involved in clinically detecting feline CE (FCE) and differentiating inflammatory from neoplastic phenotypes (2–4), monitoring FCE for progression and response to various treatment options based on clinical, clinicopathological, and histologic endpoints can be difficult (2–4). Non-invasive parameters that can be utilized as surrogate biomarkers to evaluate such clinical endpoints are desirable but have not been studied extensively in FCE (2, 5–7).

Fecal calprotectin is widely used to detect chronic intestinal inflammation in humans (8–10) and has shown promise as a non-invasive biomarker in dogs with CE (11, 12). It is a useful surrogate marker to diagnose and monitor human patients with inflammatory bowel diseases (IBD), mainly comprised of Crohn’s disease and ulcerative colitis (13, 14). In dogs, fecal calprotectin could potentially predict the response to treatment during the induction phase of treatment (11). However, fecal calprotectin has only been reported in one study on FCE (15) evaluating this biomarker at the time of diagnostic evaluation.

While fecal calprotectin concentrations were shown to poorly distinguish between cats with chronic inflammatory enteropathy (CIE) and those cats with intestinal lymphoma (15), its potential ability to predict outcomes or disease course and subclass based on treatment response has not yet been investigated in cats. Thus, this study aimed to measure fecal calprotectin concentrations in a group of cats satisfying the clinical criteria for diagnosing FCE before and during treatment. We hypothesized that fecal calprotectin concentrations would be increased in affected cats that are naïve to treatment, decrease with successful treatment comprised of immunomodulation (immunosuppressive-responsive enteropathy, IRE) and/or dietary intervention (food-responsive enteropathy, FRE), and correlate with patient characteristics that are currently interpreted as evidence of disease severity.

## 2 Method

### 2.1 Study population

Fecal samples were collected from 35 cats. These included 17 cats with FCE and 18 healthy controls. Of the 17 cats with FCE, 12 cats were retrospectively classified as FRE. This was based on an assessment over 6–230 weeks (median: 6 weeks) of exclusive feeding of an appropriate therapeutic diet. Four of the FRE cats were classified as IRE following an assessment of 6–135 weeks (median: 41 weeks) of treatment with immunomodulatory monotherapy (*n* = 3) or combination therapy (*n* = 1). One cat that did not respond to dietary intervention within the first 6 weeks was lost to follow-up and could not be classified as either FRE or IRE. Some of the cats had been included in previously published studies (16, 17). Ten cats were recruited at the Department for Small Animals at the University of Leipzig, 21 at the Queen Mother Hospital for Animals (QMHA) at the Royal Veterinary College and 5 at the University of Oxford (only control cats owned by staff members). All cats with FCE had undergone investigations, including a thorough patient history, physical examination, and clinicopathological testing. Clinical disease severity was graded using the feline chronic enteropathy activity index (FCEAI) scoring system (18); the criterion “endoscopic lesions” was omitted from the calculation of the FCEAI in cases not undergoing endoscopic evaluation. Fecal consistency was graded using the Waltham feces scoring system (19). Abdominal ultrasonography reports were available from 15 cats with FCE. Gastrointestinal biopsies were performed in 6 FCE cats; 5 cats underwent an esophagogastroduodenoscopy combined with ileocolonoscopy in 1 cat and colonoscopy in another cat. One cat had surgical biopsies obtained from the duodenum/proximal jejunum and ileum.

Healthy control cats were included based on a non-invasive assessment of health, including the absence of owner-reported clinical signs and a normal physical examination in cats that were not veterinary staff-owned.

Ethical approval for including cats from the United Kingdom was obtained from the Clinical Research Ethical Review Board at the Royal Veterinary College (#URN 2022–2123-3). Written consent was obtained for client-owned cats with chronic enteropathy recruited at the Royal Veterinary College. For staff-owned control cats recruited at the Royal Veterinary College and University of Oxford, verbal consent was obtained prior to inclusion into the study. For the cats recruited at the University of Leipzig, ethics approval was waived given that fecal samples were collected, and owners approved the use of anonymized patient data and surplus sample materials from their cat on the standard hospital admission form of the Department for Small Animals at the University of Leipzig College of Veterinary Medicine in addition to verbal consent for follow-up data.

### 2.2 Sample collection and analysis

Single spot fecal samples were collected after natural defecation from 12 cats with FCE prior to treatment, all 17 FCE cats at least 6 weeks after initiation of treatment, and all 18 healthy control cats. After collection into a sampling container, fecal samples were frozen at-20°C as soon as possible and for a maximum of 45 months. Samples were then thawed and extracted at a 1:500 dilution using the Calex® Cap device (Bühlmann Laboratories, Schönenbuch, BL, Switzerland) as described (20). Fecal extracts were centrifuged at 1,500 × *g* for 5 min, and the supernatant was assayed using the fCal® turbo particle-enhanced immunosorbent assay (PETIA) on a Roche Cobas 311 chemistry analyzer as previously validated for use with fecal samples from cats (20). The working range of this PETIA ranges from 3 to 2,000 μg/g, and the reference interval for fecal calprotectin in cats using this assay has been established as <64 μg/g (20).

### 2.3 Data analysis

Data were tested for normality using a Shapiro–Wilk test. Summary statistics are reported as medians and ranges or counts and percentages. Non-parametric two-group comparisons were performed using a Mann–Whitney *U* test (unpaired data) or a Wilcoxon signed-rank test (paired data). Pairwise correlations were analyzed by calculating a Spearman correlation coefficient (ρ). Commercially available statistical software packages (JMP v.13, SAS, Cary, NC, USA; GraphPad Prism v.10, Boston, MA, USA) were used for all calculations and statistical analyses. Statistical significance was set at *p* < 0.05.

## 3 Results

### 3.1 Study population

Patient demographics were not significantly different between cats with FCE and healthy controls (Table 1). Clinical signs in cats with FCE were reported to have been present for 1–120 months (median: 12 months) and included vomiting (14/17 cats, 82%), weight loss (11/17 cats, 65%), diarrhea (8/17 cats, 47%), reduced activity (7/17 cats, 41%), and/or hyporexia (7/17 cats, 41%). FCEAI scores at first presentation and sampling ranged from 1 to 10 (median: 5) (Table 2). Concurrent dermatological signs were present in 3/15 FCE cats (20%), and an association of clinical signs with stress was reported for 3/16 cats (19%) with FCE. Hematochezia, but not melena, was detected in 3/16 cats (19%). Prior to presentation, most cats (13/17; 76%) were fed a commercial non-therapeutic diet, whereas 4 cats (24%) had been fed a therapeutic hydrolyzed protein diet (Purina ProPlan Veterinary Diets HA dry food: *n* = 3, Hill’s Prescription diet z/d dry and tinned food: *n* = 1). Information on cohabitation was available for 14 FCE cats, of which 7 cats (50%) were from a multi-cat household and 7 cats (50%) had no feline housemates.

**Table 1:** Study population characteristics.

Variable	FCE (*n* = 17)	Healthy (*n* = 18)	*p*
Age, in years	6 (2–17)	5 (0.6–15)	0.4066
BCS, out of 9^1^	5 (3.5–6)	5 (4.5–5.5)	0.8264
Sex, female/male	7 / 10	6 / 12	0.6312
Breed			
DSH	13 (76%)	9 (50%)	0.1019
non-DSH	4 (24%)	9 (50%)	

1 Available from 18 cats. BCS, body condition score; DSH, Domestic shorthair; FCE, feline chronic enteropathy.

**Table 2:** Clinical scores and fecal calprotectin concentrations (medians and ranges) at both sampling time points in cats with FCE based on dietary response.

	Response to diet (FRE)		No response to diet (non-FRE) (includes some cats responsive to immunomodulatory treatment [IRE])	
Variable	Pre tx	Post tx	Pre tx	Post tx
n	12		5	
FCEAI score^1^	4.5 (1–9)	0	7 (1–10)	0
Waltham fecal score^2^	3.5 (3–4)	2 (2–3)	–	2
Fecal calprotectin^3^	45 (3–1,066)	16 (3–679)	314 (3–355)	3 (3–156)
Increased fecal calprotectin	4/9 (44%)	3/12 (25%)	2/3 (67%)	1/5 (20%)

1 Available from 17 cats (pre tx) and 6 cats (post tx).

2 Available from 2 cats (pre tx) and 6 cats (post tx).

3 Available from 12 cats (pre tx) and 17 cats (post tx). FCEAI, feline chronic enteropathy activity index; FRE, food-responsive enteropathy; Pre tx, before treatment; Post tx, after treatment initiation.

Clinicopathological findings in the cats with FCE are summarized in Table 3. Fasting bile acid concentration was measured in 2 cats, both measuring within the normal reference interval. Of the cats with FCE, 7 cats were tested for *Giardia* (all negative) and 3 of the cats were tested for *Tritrichomonas blagburni* (all tested negative). Fecal parasitology was negative in 5 tested cats; 10 cats were previously dewormed without performing fecal parasitology. Serum cobalamin was measured in 15 CE cats, of which 3 cats (20%) had a serum cobalamin concentration within the normal reference interval, 8 cats (53%) were hypocobalaminemic, and 4 cats (27%) were hypercobalaminemic. Three cats had received prior supplemental cobalamin (serum cobalamin concentrations: 927–1,201 pmol/L). Feline retrovirus status (FeLV/FIV) was tested in 5 cats, of which one cat was FIV-positive.

**Table 3:** Clinical and clinicopathological findings in cats with FCE (*n* = 17) prior to treatment.

Variable	Median (range) or n (%)	Unit	Reference interval
*Hematology*			
Leukocytosis	3 (18%)		
Anemia	1 (6%)		
*Blood biochemistry*			
Albumin	32.9 (25.1–36.7)	g/L	27–44
Hypoalbuminemia	3 (18%)		
Total protein	71 (63.8–80.0)	g/L	59–87
Hyperproteinemia	0		
Calcium	2.4 (2.1–3.6)	mmol/L	2.2–2.9
Hypocalcemia	2 (12%)		
Hypercalcemia	1 (6%)		
Phosphate	1.42 (1.03–1.8)	mmol/L	0.8–1.9
Hyperphosphatemia	0		
Blood urea nitrogen (BUN)	8.8 (6.5–28)	mmol/L	5.7–13.5
increased BUN	3 (18%)		
ALT activity	60 (26–470)	U/L	22–84
increased ALT activity	3 (18%)		
ALP activity	27 (14–248)	U/L	12–73
increased ALP activity	2 (12%)		
Fructosamine^a^	190 (173–247)	μmol/L	<340
increased Fructosamine	0		
Total thyroxine^b^	24.5 (9.8–55.5)	nmol/L	10–60
*Gastrointestinal panel*			
Cobalamin^c^	1,001 (149–1,201)	pmol/L	199–984
Hypocobalaminemia	3 (20%)		
Hypercobalaminemia	8 (53%)		
Folate^d^	22.8 (10.7–74.6)	μg/L	9.7–21.6
Hypofolatemia	0		
Hyperfolatemia	7 (54%)		
Pancreatic lipase (Spec fPL)^c^	2 (0.7–8.4)	μg/L	<3.5
Increased Spec fPL	2 (22%)		
Trypsin-like immunoreactivity^e^	27 (2–118.5)	μg/L	12–82

a Available from 5 cats.

b Available from 11 cats.

c Available from 15 cats.

d Available from 13 cats.

e ALT, alanine aminotransferase; ALP, alkaline phosphatase; FCE, feline chronic enteropathy.

Diagnostic imaging findings included ultrasonographic thickening of the intestinal wall in 9/15 cats (60%), increased thickness of the muscularis layer in 8/15 cats (53%), intraabdominal lymphadenopathy in 5/15 cats (33%), and evidence of ascites in 1/15 cats (7%); loss of intestinal wall layering was not seen in any cat. Endoscopy was performed in 5 cats, with lesion scores in the stomach ranging from 0 to 1 (median: 0; *n* = 5), in the duodenum/proximal jejunum from 0–1 (median: 1; *n* = 6), in the ileum from 0 to 1 (median: 1; *n* = 4), and in the colon from 0–1 (median: 0.5; *n* = 4); linear erythematous lesions were not detected in any of the cats. Histopathology revealed inflammatory and structural changes in the tissue biopsy specimens evaluated, with WSAVA Gastrointestinal histopathology scores ranging from 0 to 3 (median: 1) for the stomach, 1–11.5 (median: 1.5) for the duodenum/proximal jejunum, 1–3 (median: 2) for the ileum, and 1–4 (median: 2.5) for the colon; one cat had immunohistochemistry for CD3 and CD20 performed that was not suggestive of lymphoma. A strong eosinophilic component of the inflammatory infiltrate was seen in 1 cat.

Of the 12 cats classified as FRE, 7 cats showed a complete response to a therapeutic hydrolyzed protein diet, 2 cats responded completely to a therapeutic limited-ingredient novel protein diet, and 3 cats were completely responsive or showed a near-complete response to a therapeutic gastrointestinal diet. All 4 cats with IRE received prednisolone, which was given in combination with chlorambucil in 1 cat, and 3 of the cats were also fed a therapeutic hydrolyzed protein diet. Additional treatment consisted of prebiotics/probiotics, antiemetics, appetite stimulants, folate and/or cobalamin supplementation, or a combination. Fecal samples following treatment were collected 6–230 weeks (median: 6 weeks) after initiation. Long-term follow-up was available from 6 cats, all still alive at 12–45 months (median: 24 months) after the last fecal sample collection.

### 3.2 Fecal calprotectin concentrations

Fecal calprotectin concentrations prior to treatment were significantly higher in cats with FCE (median: 61 μg/g, range: 3–1,066 μg/g, *n* = 12) than in healthy controls (median: 6 μg/g, range: 3–193 μg/g, *n* = 18; *p* = 0.0235), whereas fecal calprotectin concentrations after treatment initiation were not significantly different (median: 15 μg/g, range: 3–679 μg/g, *n* = 17) from healthy (*p* = 0.8112). Cats that presented already on but were not completely responsive to a hydrolyzed protein diet (non-responders, mostly IRE cases) had numerically higher fecal calprotectin concentrations (median: 335 μg/g, range: 3–785 μg/g, *n* = 4) than cats fed a commercial non-therapeutic diet (median: 37 μg/g, range: 3–1,066 μg/g, *n* = 8), but the difference was not statistically significant (*p* = 0.4962).

Fecal calprotectin concentration prior to treatment was moderately correlated with the FCEAI score (*ρ* = 0.54, *p* = 0.0316) and correlated inversely with the serum total calcium concentration (*ρ* = −0.84, *p* = 0.0006) but not with any other clinicopathological parameter. There was also no correlation between fecal calprotectin concentrations and Waltham fecal scores before (*p* = 0.6816) or after initiation of treatment (*p* = 0.0705). The effect of hematochezia on fecal calprotectin concentrations could not be evaluated due to the small number of cats with this characteristic. The number of cats with gastrointestinal tissue biopsies was also too few to assess for possible associations between criteria of mucosal disease severity and fecal calprotectin concentrations. However, compared to all other cats with biopsies revealing a lymphoplasmacytic inflammatory infiltrate, two cats had a neutrophilic component to the duodenal/jejunal and/or ileal mucosal infiltrate. These two cats showed a complete response to dietary intervention and had the highest fecal calprotectin concentrations at the time of first sampling (1,066 μg/g and 785 μg/g, decreasing to 679 μg/g and 111 μg/g under treatment). Although the numbers were small, fecal calprotectin concentrations before treatment correlated with the severity of inflammatory histologic lesions across all gastrointestinal segments investigated (*ρ* = 1.00, *p* < 0.0001; *n* = 3).

**Figure 1:**
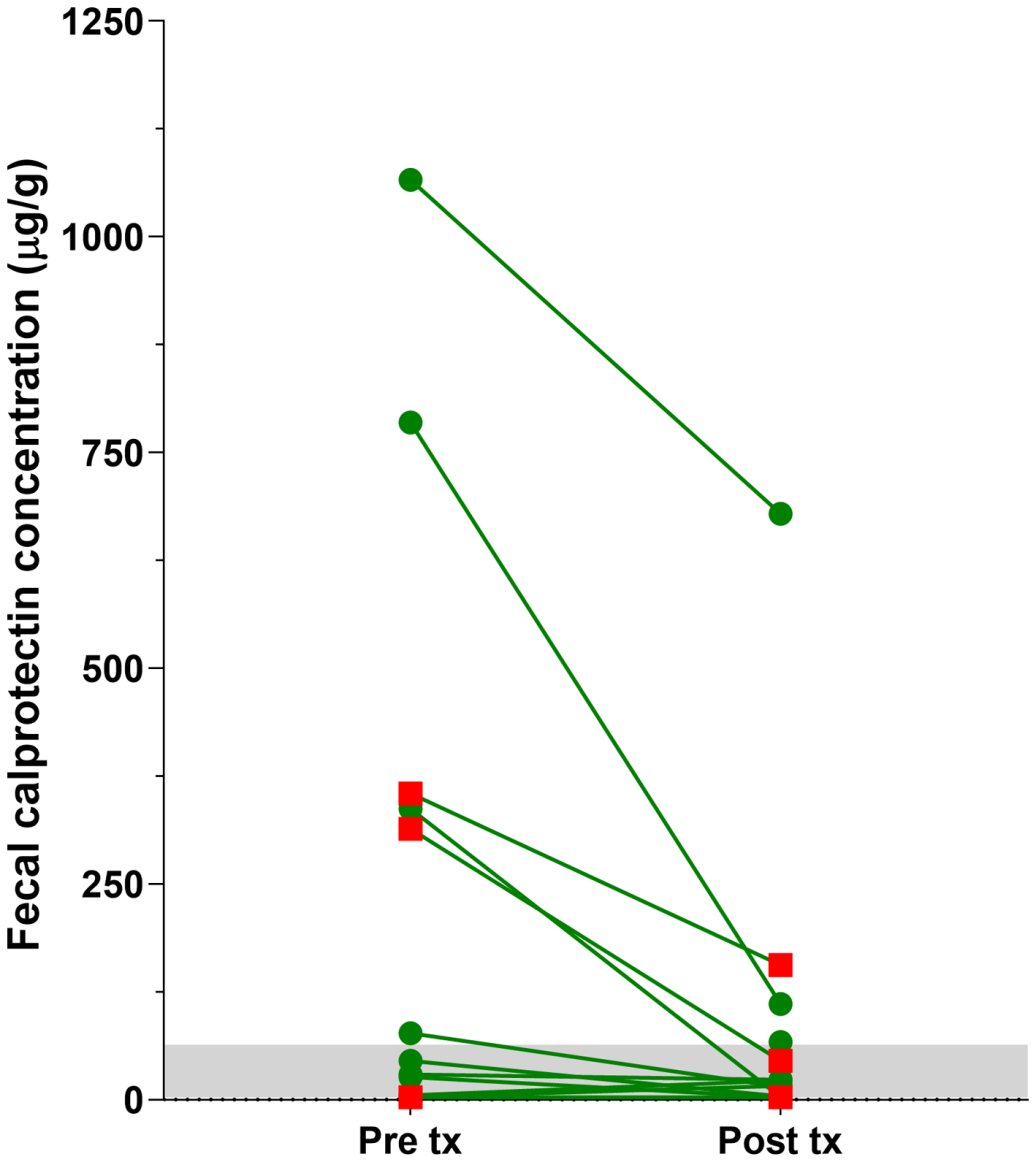
Fecal calprotectin concentrations in cats with FCE prior to (pre tx; median: 61 μg/g, range: 3–1,066 μg/g, *n* = 12) and after treatment initiation (post tx; median: 15 μg/g, range: 3–679 μg/g, *n* = 17). Concentrations of calprotectin decreased significantly between both collection time points (*p* = 0.0098) but this could not be separately analyzed statistically for cats classified as IRE (red squares) compared to those responding to dietary intervention alone (green dots). The gray shaded area indicates the normal reference interval for feline fecal calprotectin concentrations (<64 μg/g).

Fecal calprotectin concentrations decreased significantly from before to after treatment initiation (*p* = 0.0098; Figure 1) but could not be assessed for a difference between cats with FCE responding to dietary intervention (FRE) and those with no clinical response to diet due to the small number of non-responders or IRE cases with paired fecal samples (*n* = 3). Of the cats with increased fecal calprotectin concentrations (>64 μg/g) before treatment, cats classified as FRE had slightly larger decreases in fecal calprotectin concentrations from the initial measurement (median: 83%, *n* = 4) than non-responders to diet (median: 71%, *n* = 2) (Table 2). Disease duration prior to first sampling was not associated with fecal calprotectin concentrations (*p* = 0.6733) or after treatment initiation (*p* = 0.3489) nor with the response to dietary intervention (*p* = 0.3658).

Fecal calprotectin concentrations measured after treatment initiation were weakly correlated with pre-treatment serum concentrations of cobalamin (*ρ* = 0.55, *p* = 0.0346; *n* = 15) and albumin (*ρ* = −0.53, *p* = 0.0292; *n* = 17), the severity of histologic lesions in the duodenum/proximal jejunum (*ρ* = 0.83, *p* = 0.0427; *n* = 6), and with pre-treatment calprotectin concentrations (*ρ* = 0.61, *p* = 0.0338; *n* = 12). Fecal calprotectin concentrations after treatment initiation were generally lower with longer intervals between both sampling time points (*ρ* = −0.54, *p* = 0.0250).

## 4 Discussion

This study showed that fecal calprotectin concentrations are increased in cats with chronic enteropathy, as has been previously reported by our group (15). Similar to previous observations in cats (15), fecal calprotectin concentrations are generally lower in cats than in human inflammatory bowel diseases (21, 22) but comparable to those concentrations detected in dogs with CE (11, 23). This likely reflects the lack of either a pleomorphic or predominating polymorphonuclear cell population in most cases of feline and canine CIE (12) when compared to human inflammatory bowel diseases (24, 25).

Decreases in fecal calprotectin concentrations in this study were seen on varying treatment strategies, all resulting in an improvement or remission of clinical signs. Biologically relevant decreases in fecal calprotectin concentrations of at least 40% (20) were seen in all but one cat classified as FRE, with fecal calprotectin decreasing from 29 μg/g to 23 μg/g, however, both concentrations of which are within the normal reference interval. Thus, fecal calprotectin concentrations might be associated with more severe disease (clinical, histologic lesions, serum albumin and/or total calcium concentration) and therefore may present a good surrogate biomarker of disease activity. In accordance with this, is the finding that there was a moderate correlation of fecal calprotectin concentrations with FCEAI at the time of diagnosis. This further suggests a potential utility of the calprotectin test as a surrogate marker to assess disease severity, particularly as it presents a stable marker that can be measured in naturally passed fecal samples conveniently obtained by the owner at home. This correlation contrasts with our previous observation in cats with CIE and intestinal lymphoma (15) and a study evaluating a similar biomarker in the same protein subfamily (6). However, a similar moderate correlation with the clinical disease severity (particularly the severity of diarrhea) was shown for the number of mucosal S100/calgranulin-positive cell counts in FCE cases (26). In addition to the difference in the study population with varying phenotypes, it needs to be stressed that FCEAI assessment relies on owner-observed clinical signs in addition to objective laboratory findings. The lack of a correlation between fecal calprotectin concentrations and Waltham feces scores is consistent with previous results in another cohort of cats (15).

The clinical correlate of a decrease in fecal calprotectin with response to treatment could be a reduction in calprotectin-expressing phenotypes of inflammatory cells (e.g., activated infiltrating macrophages) and/or a decrease in the cellular calprotectin expression levels (26). While routine histopathological assessment of polymorphonuclear cells, which are the main mucosal sites of calprotectin expression, does not necessarily correlate with the number of cells expressing calprotectin along the gastrointestinal mucosa (26), this study found the highest fecal calprotectin concentrations in cats with FCE and a neutrophilic component of the mucosal infiltrate.

The presence and proportion of gastrointestinal clinical signs of the cats included in this study agree with those reported by others (1). Resolution of these clinical signs (i.e., a significant reduction in the FCEAI score) was considered the clinical endpoint at the second sampling under treatment. While this is the most practical and routinely selected approach to reassess cats with FCE within the scope of disease monitoring, consideration that clinical response/remission might not necessarily reflect the achievement of mucosal healing (deep remission) has been a concept in human inflammatory bowel disease for some time (27) and has been critically discussed recently for canine CE (28). This concept might also be reconsidered for FCE and might explain why fecal calprotectin concentrations remain increased (although significantly reduced from before treatment induction) in a few cats despite a clinical response. It might also explain the detection of very low fecal calprotectin concentrations (3 μg/g) in 3 of the cats diagnosed as IRE and receiving immunomodulatory treatment for 6–135 weeks (median: 76 weeks). Compared to hypoalbuminemia being detected in about 10–30% of FCE cases (3), hypocalcemia has not been reported as a feature of FCE and was also not associated with hypoalbuminemia in this study. Thus, vitamin D metabolism might also be an interesting avenue to study in FCE (29, 30).

We acknowledge that, while most cats received the same commercial therapeutic hydrolyzed protein diet between both fecal sampling time points (*n* = 10), a uniform dietary regimen was not followed prior to the first sample collection. While the nutritional strategies varied to some extent among the cats included in the study, lack of response to one diet might not reflect the response to and preclude achieving full remission with the choice of a different diet (15, 31). In line with this, long-term outcomes were not evaluated in the cats included in the study. Thus, a “lane switch” from FRE to IRE – or potentially even vice versa – as proposed by some small animal gastroenterologists cannot be excluded in these cases.

Another shortcoming of the study is that endoscopy and intubation of the ileum for tissue sampling were not performed in all cats to document and characterize inflammation and distinguish intestinal lymphoma cases in the FCE group (32, 33), particularly in those cats not responding to dietary intervention alone. However, similar to fecal S100A12 concentrations (6), fecal calprotectin concentrations are not discriminatory for these two entities of FCE (15). In addition, the non-invasive determination of health in the control group, as mandated by ethical guidelines, did not allow the assessment of potential histological findings consistent with a diagnosis of FCE in this group. Thus, it remains unknown whether the 3 cats with higher fecal calprotectin concentrations in the control group could have had subclinical intestinal disease including occult endoparasites. Lastly, storage of unextracted fecal samples at-20°C varied from a few days up to 45 weeks. While this might be associated with a slight decline in fecal calprotectin concentrations, this protein complex has previously been shown to be remarkably stable in fecal samples (34).

We conclude that fecal calprotectin concentrations decrease with successful therapeutic intervention in cats with FCE and might be a good marker to assess intestinal health initially and longitudinally in affected cats. Future studies are warranted to assess fecal calprotectin in response to mucosal healing vs. clinical response and to decipher short-term, intermediate-term, and long-term changes in fecal calprotectin concentrations in cats with FRE from those in cases of IRE, both responders (IRE) and non-responders (NRE).

## Data availability statement

The raw data supporting the conclusions of this article will be made available by the authors, without undue reservation.

## Ethics statement

The animal studies were approved by Clinical Research Ethical Review Board at the Royal Veterinary College. The studies were conducted in accordance with the local legislation and institutional requirements. Written informed consent was obtained from the owners for the participation of their animals in this study.

## Author contributions

RH: Conceptualization, Formal analysis, Funding acquisition, Investigation, Project administration, Resources, Software, Writing – original draft. DR: Conceptualization, Data curation, Formal analysis, Investigation, Writing – review & editing. IT: Data curation, Investigation, Writing – review & editing. GK: Methodology, Resources, Software, Validation, Writing – review & editing. AK: Conceptualization, Funding acquisition, Investigation, Project administration, Resources, Supervision, Writing – original draft.
